# *Saccharomyces boulardii* modulates oxidative stress and renin angiotensin system attenuating diabetes-induced liver injury in mice

**DOI:** 10.1038/s41598-021-88497-w

**Published:** 2021-04-28

**Authors:** Leticia Barssotti, Isabel C. M. E. Abreu, Ana Beatriz P. Brandão, Raquel C. M. F. Albuquerque, Fabiana G. Ferreira, Miguel A. C. Salgado, Danielle D. S. Dias, Kátia De Angelis, Rodrigo Yokota, Dulce E. Casarini, Lívia B. Souza, Carla R. Taddei, Tatiana S. Cunha

**Affiliations:** 1 Department of Science and Technology, Federal University of São Paulo (Unifesp), São José dos Campos, Brazil; 2 Department of Medicine, Federal University of São Paulo (Unifesp), São Paulo, Brazil; 3 Department of Bioscience and Oral Diagnosis, State University Julio de Mesquita Filho (Unesp), São José dos Campos, Brazil; 4 Department of Physiology, Federal University of São Paulo (Unifesp), São Paulo, Brazil; 5 Department of Clinical and Toxicological Analyses, University of São Paulo (Usp), São Paulo, Brazil; 6 Federal University of São Paulo (Unifesp) - Institute of Science and Technology, Talim, 330 – Vila Nair, São José dos Campos, SP 12231-280 Brazil

**Keywords:** Diabetes complications, Type 1 diabetes

## Abstract

Type 1 diabetes (T1DM) is a chronic disease characterized by hyperglycemia due to a deficiency in endogenous insulin production, resulting from pancreatic beta cell death. Persistent hyperglycemia leads to enhanced oxidative stress and liver injury. Several studies have evaluated the anti-diabetic and protective effects of probiotic strains in animal models. In the present study, we investigated, through histopathological and biochemical analyses, the effects of eight weeks of administration of *Saccharomyces boulardii (S. boulardii)* yeast on the liver of streptozotocin (STZ) induced diabetic C57BL/6 mice. Our results demonstrated that *S. boulardii* attenuates hepatocytes hydropic degeneration and hepatic vessels congestion in STZ-induced diabetic mice. The treatment attenuated the oxidative stress in diabetic mice leading to a reduction of carbonylated protein concentration and increased activity of antioxidant enzymes superoxide dismutase and glutathione peroxidase, compared to untreated diabetic animals. The results also show the beneficial influence of *S. boulardii* in regulating the hepatic concentration of renin angiotensin system (RAS) peptides. Therefore, our results demonstrated that *S. boulardii* administration to STZ-induced diabetic mice reduces oxidative stress and normalizes the concentration of RAS peptides, supporting the hypothesis that this yeast may have a role as a potential adjunctive therapy to attenuate diabetes-induced liver injury.

## Introduction

Type 1 diabetes (T1DM) is a chronic autoimmune disease characterized by hyperglycemia caused by a deficiency in endogenous insulin production as a consequence of pancreatic beta cell death^1–3^. Persistent hyperglycemia increases the formation of reactive oxygen species (ROS), which can occur during glucose oxidation, nonenzymatic glycation of proteins, and oxidative degradation of glycated proteins^4,5^.

The antioxidant enzyme superoxide dismutase (SOD) is responsible for degrading these oxygen-free radicals to hydrogen peroxide, which then can be converted to water by glutathione peroxidase (GPx) or to water and oxygen by catalase (CAT)^6^. In diabetic individuals, the formation of ROS combined with a decrease in the antioxidant defense leads to an increase in oxidative stress, which in turn can cause oxidation of proteins and lipids, excessive activation of pro-inflammatory pathways and DNA damage. In the liver, these cell injuries are associated with hepatocyte necrosis and apoptosis^7–9^.

Renin angiotensin system (RAS) is a key regulator of vascular resistance, sodium and water homeostasis, and has been strongly implicated in the pathophysiology of diabetic complications, including pancreatic beta cell dysfunction, renal and cardiovascular disease^10^. Whereas the circulating RAS is usually normal or suppressed in T1DM, hyperglycemia activates local RAS, increasing renin activity and the synthesis of the main RAS peptide, angiotensin II (Ang II) in renal^11^, cardiac^12^ and pancreatic cells^13^, which in turn stimulates ROS generation. In the liver, Ang II can be secreted by activated hepatic stellate cells after liver damage, and plays a critical role during liver disease pathogenesis, amplifying oxidative stress^14,15^. Also, the local RAS might affect tissue angiogenesis, proliferation, cell growth, apoptosis, tissue inflammation and fibrosis, stimulating insulin resistance, de novo lipogenesis and mitochondrial dysfunction, especially during injury^15^.

On the other hand, it is well known that the maintenance of intestinal microbiota influences metabolic health and inflammatory processes^16^, and during metabolic imbalance, probiotics also regulate oxidative stress^17^. *Saccharomyces boulardii (S. boulardii)* is a non-pathogenic yeast that was first isolated from the lychee fruit in Indochina and used to treat diarrhea in the early 1950s^18^. Recent studies have shown a beneficial effect of this probiotic in the context of metabolic disorders, and inflammatory conditions originated from intestinal infections or liver damage^19–21^.

Our research group has already demonstrated that administration of *S. boulardii* in streptozotocin (STZ) induced diabetic mice improves the cardiac inflammatory profile and attenuates autonomic cardiovascular dysfunction, modulating several pathophysiological mechanisms^22^. Additionally, our group demonstrated the beneficial effects of *S. boulardii* on gut microbiota composition and its capacity to improve hepatic inflammatory and metabolic profile in the experimental model of T1DM^23^. In the present study, we investigated the effect of *S. boulardii* administration on the liver injury of STZ-induced diabetic mice as well as its role in the modulation of hepatic RAS peptides and oxidative stress markers.

## Methods

### Animals

C57BL/6 male mice (15–20 g, 5 weeks old) were purchased from CEDEME (Federal University of São Paulo—UNIFESP, Brazil). Mice were maintained in the animal facility of the Department of Science and Technology (ICT/UNIFESP—São José dos Campos, Brazil). Animals had free access to commercial diet and water and were kept at a room temperature of 22 ± 2 °C under a 12-h light–dark cycle. Mice were randomly allocated into four groups: Control (C), Control + Probiotic (CP), Diabetes (D), and Diabetes + Probiotic (DP) (n = 6–9/group). All animal studies were approved by the Institutional Ethics Committee on Use of Animal (CEUA-UNIFESP/Protocol #2898091216) and were conducted in accordance with guidelines established by the National Council of Control of Animal Experimentation (CONCEA)^23^. The present study was performed in compliance with the principles of the ARRIVE (Animal Research: Reporting of In Vivo Experiments) guidelines^24^.

### Experimental design

Mice (six-week-old) from D and DP groups received a single intraperitoneal injection of STZ (150 mg/kg, Sigma Chemical Co., St. Louis, MO, USA) dissolved in 0.01 M citrate buffer at pH 4.5^22,23^. C and CP groups injection consisted of an equivalent volume of citrate buffer. After the administration of STZ, the animals were given a 5% glucose solution to prevent hypoglycemia, for 24 h. Single STZ high dose exerts direct toxicity on beta cells, which results in necrosis within 48–72 h and permanent hyperglycemia^25,26^.

Four days after the STZ injection, blood samples were collected through the tail vein, and blood glucose level was determined (Accu-Check Advantage Blood Glucose Monitor—Roche Diagnostic Corporation, Indianapolis, IN). Mice with blood glucose ≥ 250 mg/dl after fasting for four hours were considered diabetic. During eight weeks, mice from CP and DP groups received a daily dose (0.5 × 10^8^ colony-forming units, CFU) of *S. boulardii,* THT 500,101 strain (THT: Probiotics and Starters Cultures, Belgium) dissolved in 0.3 ml of sterile water, by oral gavage^22,23^. The control groups received the same volume of sterile water. After eight weeks of treatment, animals were euthanized by decapitation.

The liver was rapidly excised, precisely dissected, and weighed. Caudate lobe was stored at − 80 °C for further analyses, and the median lobe was fixed in 10% buffered formalin for histological analyses. Trunk blood was collected in ice-chilled heparinized tubes and blood was centrifuged at 10,000×g for 10 min at 4 °C. Serum was separated, aliquoted and stored at − 80 °C for further analyses.

### Histological measurements

#### Hepatocytes area

For morphometric analysis of Hematoxylin & Eosin stained sections (400 × magnification), the hepatocyte perimeters of three random regions of the tissue were determined freehand by one blinded reader, and 500 to 1100 hepatocytes per group were measured. We carefully selected the tissue sections, avoiding cells around hepatic veins or in the edges of the slices, since they might present distortions. To avoid any uncertainty measurement, we included a high number of cells in the analysis and also used the three sections from each slide. Digital photomicrographs were captured using the software Pannoramic Viewer (3DHISTECH Ltd.) and the number of hepatocytes per area from four random sections of each sample was computed.

#### Collagen content

Six hundred images of stained sections with Picrosirius Red were captured by Olympus Bx43 microscope connected to a digital camera Q-Color 3 Olympus. The images were digitalized at 2048 × 1536 pixels, 24 bit/pixel of resolution with a global magnification of 100 × by cellSens Standard Olympus. The quantitative evaluation of the area occupied by collagen deposition over the total area of liver tissue was performed using the image analysis software CellProfiler 3.1.5^27^, according to a pipeline adapted from Sant’anna, Sant’anna, and Parolini^28^.

#### Hepatic vascular congestion

To determine the degree of vascular congestion, hepatic vessels from Hematoxylin & Eosin stained sections (200 × magnification) were counted and classified as (1) no congestion, (2) slight congestion (less than half of the vessels full of blood cells), (3) moderate congestion (about half of the hepatic vessels presenting signs of congestion) and severe congestion (more than half of the vessels filled up with blood cells). The results were presented as a percentage of the total number of analyzed blood vessels.

### Determination of serum ALT and AST levels

The alanine aminotransferase (ALT) and aspartate aminotransferase (AST) activities were determined to investigate liver damage using commercially available kits (Labtest Diagnostica S/A, Lagoa Santa, MG, Brazil) according to the manufacturer’s instructions.

### Preparation of liver homogenate

On the day of extraction, a frozen liver fragment was homogenized for 30 to 45 s, in a cooled 2 ml microcentrifuge tube, with stainless steel beads in it. For this, ice-cold phosphate buffer 30 mM and potassium chloride pH 7.4, containing sucrose (240 mM), NaCl (300 mM), and the following protease inhibitors: potassium EDTA (25 mM), *o*-phenanthroline (0.44 mM), pepstatin A (0.12 mM), 4-chloromercuribenzoic acid (1 mM), phenylmethanesulfonyl fluoride (PMSF) 100 mM and protease inhibitor cocktail (1 tablet/10 ml extraction solution, cOmplete Mini Protease Inhibitor, Roche, USA), were added to the microcentrifuge tube in a proportion 10 ml per gram of tissue and used for homogenate preparation. Samples were centrifuged at 4000 rpm for 10 min and the supernatant was stored at 80 ºC for biochemical analyses.

### Protein oxidation

Protein oxidation was determined through a reaction of protein carbonyl groups with 2,4-dinitrofenylhydrazyne to form 2,4-dinitrophenylhydrazone, which can be measured spectrophotometrically at 360 nm^29^. The reaction medium consisted of guanidine 6 M in hydrochloric acid 2.5 M at pH 2.5, 2,4 dinitrophenylhydrazine in hydrochloric acid 2.5 M, trichloroacetic acid (TCA) 20%, TCA 10% and ethanol-ethyl acetate 1:1 (v/v).

### Chemiluminescence induced by tert-butyl hydroperoxide (tBOOH)

The chemiluminescence was measured by a beta counter (TriCrab 2800TR, PerkinElmer) in a darkroom. The reaction medium consisted of a phosphate-buffer solution 20 mmol/l (with potassium chloride 140 mmol/l, pH 7.4) and tert-butyl hydroperoxide 400 mmol/l^30^.

### Antioxidant enzyme activity

The SOD activity was evaluated by the inhibition of the reaction between peroxide anion and pyrogallol. The determination of CAT activity was by monitoring the decrease in hydrogen peroxide (H_2_O_2_) absorbance at 240 nm^31^. GPx activity was determined by adding glutathione reductase, glutathione and, later on, nicotinamide adenine dinucleotide phosphate (NADPH) and monitoring the alteration of absorbance at 340 nm^32^.

### Angiotensin quantification

The samples were prepared for solid-phase extraction with the addition of the Sar1-Leu8 Ang II internal standard and thus extracted on Sep-PakC18 3 cc columns (Waters, USA). Extraction control was done using a tenfold concentration with the peptides of interest: angiotensin I (Ang I), Ang II, angiotensin 1–7 (Ang 1–7), angiotensin 1–5 (Ang 1–5), angiotensin 1–9 (Ang 1–9), angiotensin III (Ang III) and angiotensin 3–7 (Ang 3–7) (Sigma, USA). The peptides were separated on a Luna C18 reverse-phase column (100 × 2.0 mm, 3 μm, Phenomenex, USA) using a linear gradient of mobile phase A (H_2_O, ammonium acetate and formic acid) and mobile phase B (methanol, ammonium acetate, and formic acid) in Agilent 1260 HPLC (Agilent, USA), and subsequently injected into the Mass Spectrometer coupled to HPLC, AB Sciex 5500 QTRAP (Sciex, USA). The peptides' detection was performed with the multiple monitoring reaction (MRM) according to their mass/load (m/z) and retention. Calculations were performed from a calibration curve (with matrix) in the range of 0.5 fmol to 200 fmol per injection. Quantification of the samples was determined by calculating the peak area displayed for the mass of the target ions in the spectrometer, referenced to the known amount of standard peptides acquired under the same conditions over the same time. The results were analyzed using Multiquant 3.0.3 software (Sciex, USA). The results were expressed as fmol/mg.

### Statistical analysis

For this study, the sample size was calculated based on the total of proposed experimental groups (4), the standard deviation of blood glucose (58 mg/dl), and the difference in blood glucose measurement between the control and the STZ-induced diabetic group (241 mg/dl); α = 0.05 and 95% statistical power were considered^33^. Statistical analysis was performed using GraphPad Prism. The Kolmogorov–Smirnov algorithm was used to determine whether each variable had a normal distribution. Parametric results were evaluated with Two-way ANOVA followed by Tukey test for multiple comparisons of means, expressed as mean ± standard error of the mean, and nonparametric results were evaluated with Kruskal–Wallis, followed by Dunn’s multiple comparisons test, expressed as median ± IQR (interquartile range). Differences were considered statistically significant when p ≤ 0.05.

## Results

### Blood glucose and body weight

Before treatment, diabetic mice presented significantly lower body weight compared with C and CP groups (C = 19.30 ± 0.51 g, CP = 19.07 ± 0.56 g vs. D = 13.90 ± 0.38 g, DP = 15.80 ± 0.37 g). The difference remained until the end of the eight-week protocol, and probiotic did not affect this parameter (C = 22.16 ± 0.58 g, CP = 22.31 ± 0.50 g vs. D = 14.54 ± 0.91 g, DP = 16.90 ± 0.46 g).

**Figure 1:**
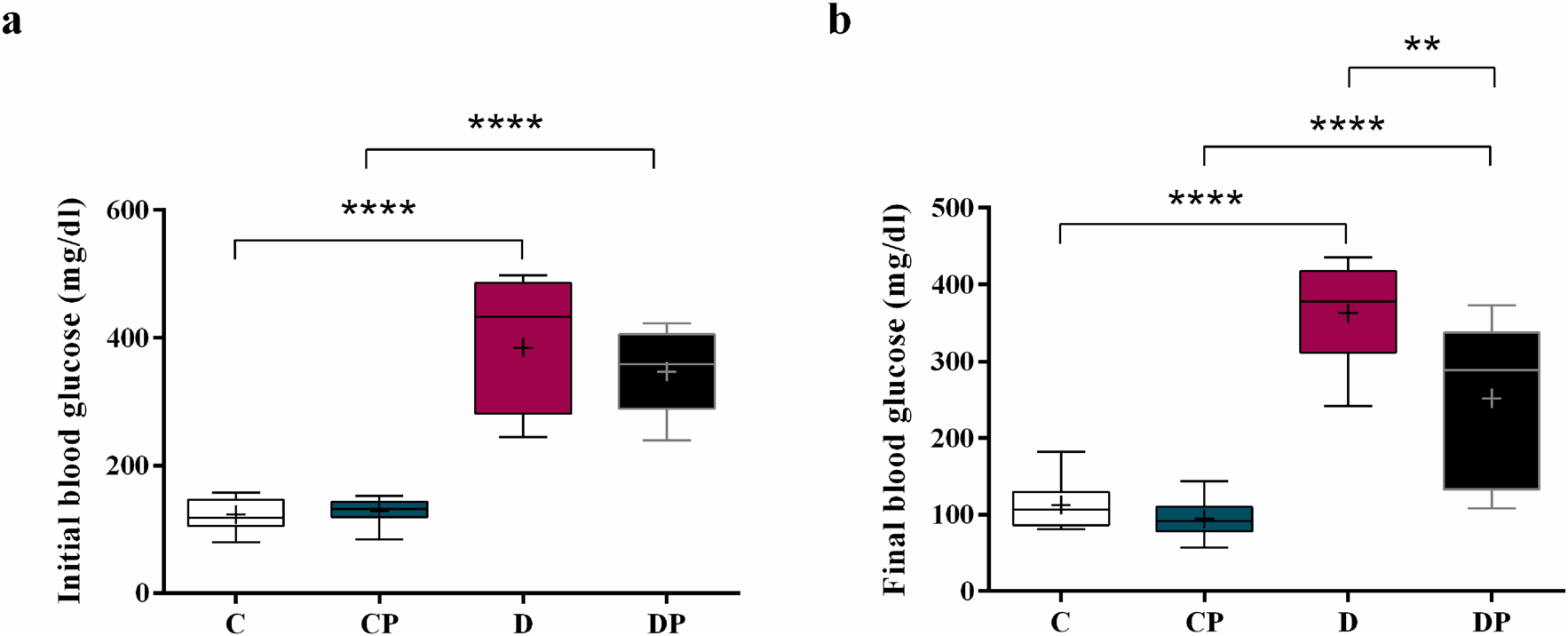
Fasting blood glucose in mg/dl **(a)** at the beginning and **(b)** at the end of the protocol (week 8). Control (C), control + probiotic (CP), diabetic (D) and diabetic + probiotic (DP) groups (n = 6–9/group). Data are from C57BL/6 STZ-induced diabetic mice, + /− *Saccharomyces boulardii* (0.5 × 10^8^ colony-forming units, THT 500,101 strain*,* Probiotics and Starters Cultures, Belgium) for 8 weeks. A horizontal line inside the box indicates the median value of the samples within each group, and the upper and lower edges of the box indicate the quartiles. The significance was determined by Two-way ANOVA followed by Tukey test for multiple comparisons of means (GraphPad Prism 6). + indicates mean value. **p ≤ 0.01, ****p ≤ 0.0001.

After diabetes induction, mice with blood glucose levels higher than 250 mg/dl were considered diabetic (Fig. 1a). The diabetic group that received a daily dose of *S. boulardii* presented a 30% reduction of blood glucose compared to the untreated group (DP = 251.9 ± 36.40 mg/dl vs. D = 362.9 ± 21.26 mg/dl), showing the beneficial effect of the probiotic on glucose homeostasis (Fig. 1b).

### Histological analyses

#### Hepatocyte area and collagen content

**Figure 2:**
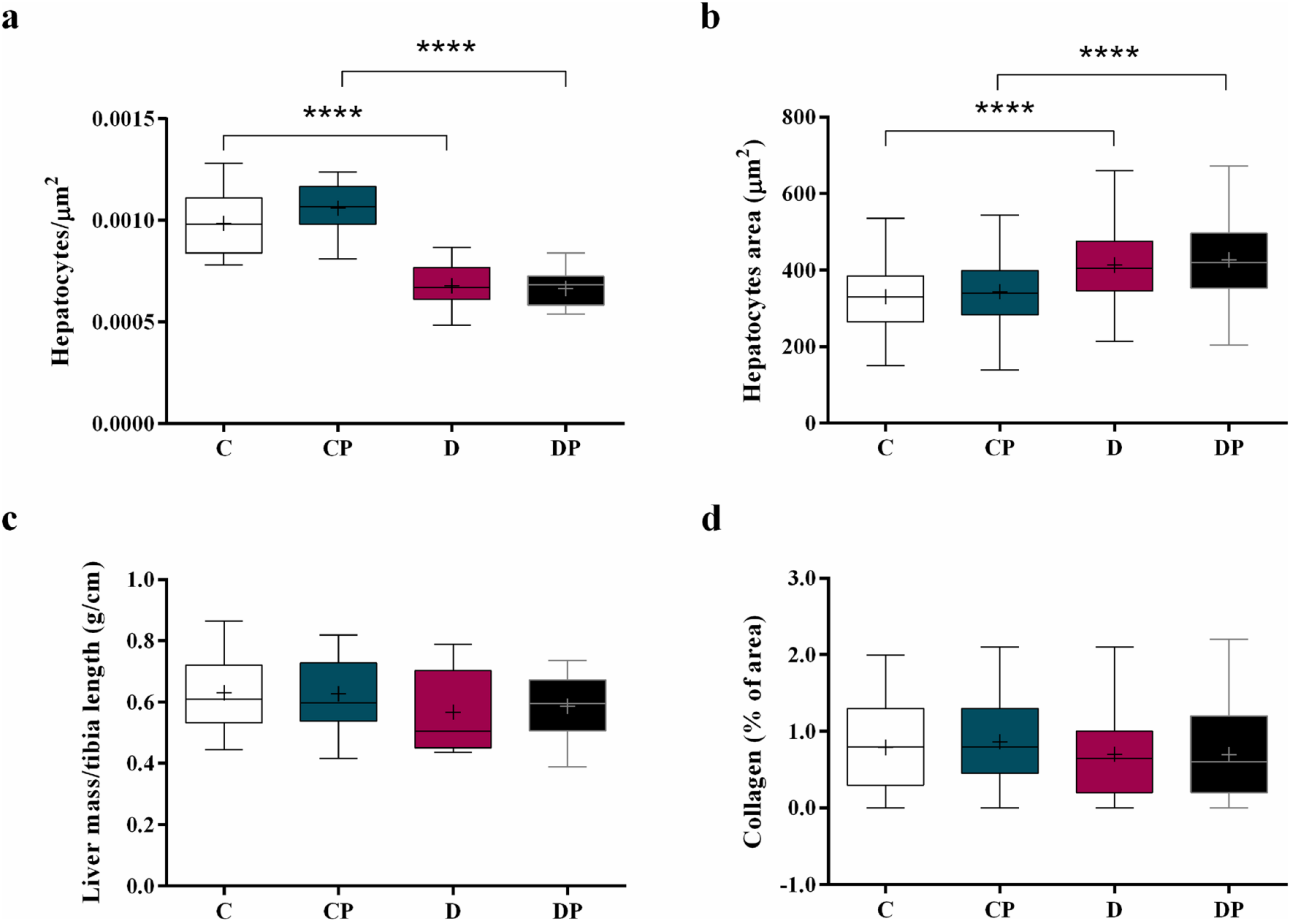
**(a)** Number of hepatocytes per µm^2^ **(b)** hepatocytes area in µm^2^, **(c)** liver mass corrected by tibia length (g/cm), **(d)** collagen content (%) analyzed using CellProfiler 3.1.5 in control (C), control + probiotic (CP), diabetic (D) and diabetic + probiotic (DP) groups (n = 6–9/group). Data are from C57BL/6 STZ-induced diabetic mice, + /− *Saccharomyces boulardii* (0.5 × 10^8^ colony-forming units, THT 500,101 strain*,* Probiotics and Starters Cultures, Belgium) for 8 weeks. A horizontal line inside the box indicates the median value of the samples within each group, and the upper and lower edges of the box indicate the quartiles. For **(b,d)** significance was determined by Kruskal–Wallis followed by Dunn’s test, and for **(a,c)** significance was determined by Two-way ANOVA followed by Tukey test for multiple comparisons of means (GraphPad Prism 6). + indicates mean value. **** p ≤ 0.0001.

The number of hepatic cells per µm^2^ in D and DP groups decreased (Fig. 2a) (D = 0.0007 hepatocytes/µm^2^, DP = 0.0007 hepatocytes/µm^2^ vs. C = 0.0010 hepatocytes/µm^2^, CP = 0.0011 hepatocytes/µm^2^), and the hepatocytes area increased (Fig. 2b) (D = 404.7 ± 130.2 µm^2^, DP = 419.4 ± 143.4 µm^2^ vs. C = 329.6 ± 120.0 µm^2^, CP = 339.8 ± 115.5 µm^2^ ) compared with C and CP groups. However, the liver mass (Fig. 2c) was not affected by diabetes or probiotic. The collagen content data (analyzed from the histological images of hepatic tissue with 100 × magnitude) did not significantly change (Fig. 2d).

#### Hepatic vascular congestion

**Figure 3:**
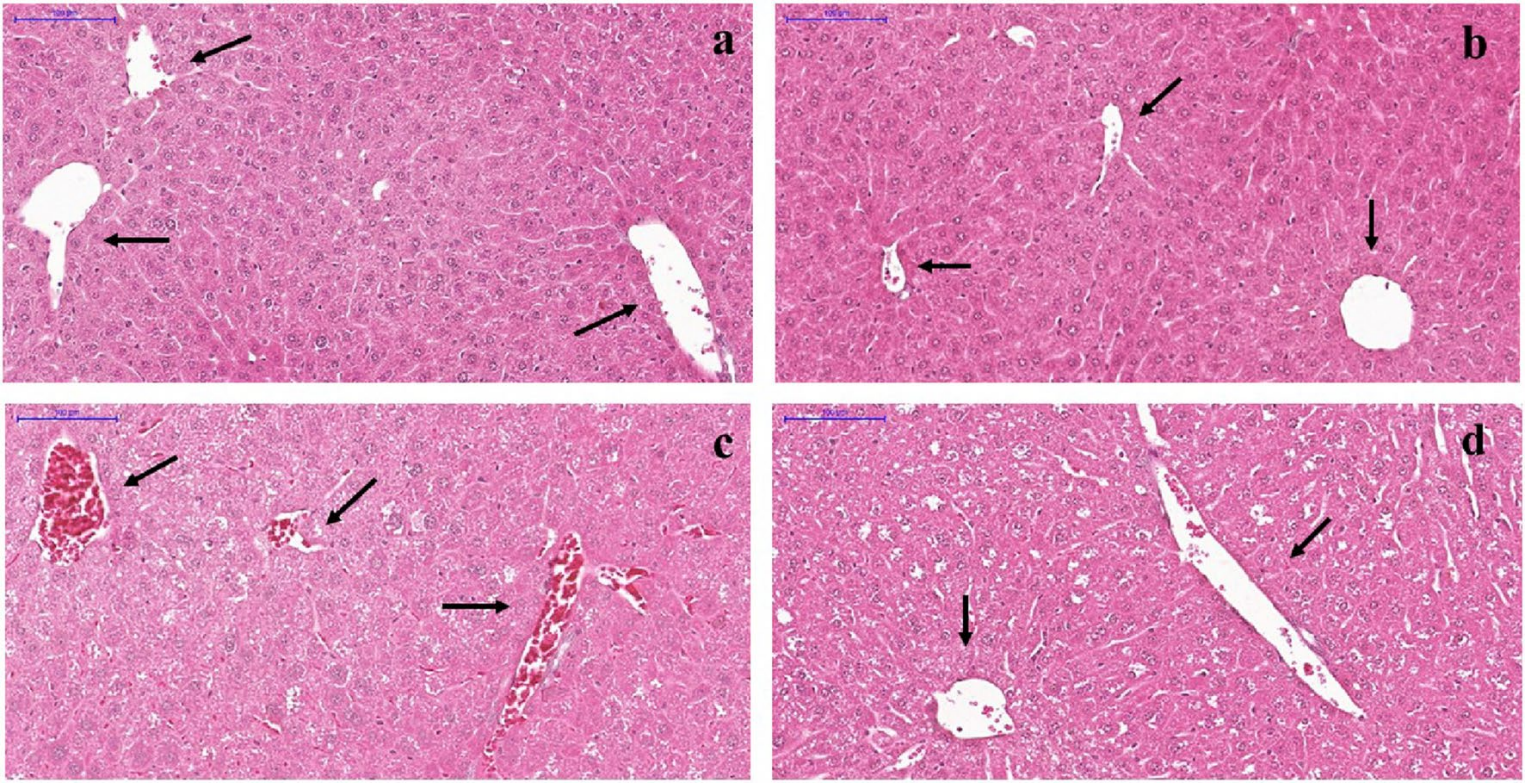
Representative photomicrographs of Hematoxylin & Eosin stained liver from C57BL/6 STZ-induced diabetic mice, + /− *Saccharomyces boulardii* (0.5 × 10^8^ colony-forming units, THT 500,101 strain*,* Probiotics and Starters Cultures, Belgium) for 8 weeks. Control **(a)** and control + probiotic **(b)** show normal hepatocytes organization and hepatic vessels (→) with no sign of congestion. Hepatic vessels ( →) from the diabetic group **(c)** showed severe congestion while diabetic mice + probiotic **(d)** showed slight vessel congestion similar to control. Scale bar: 100 μm; 200 × magnification.

**Figure 4:**
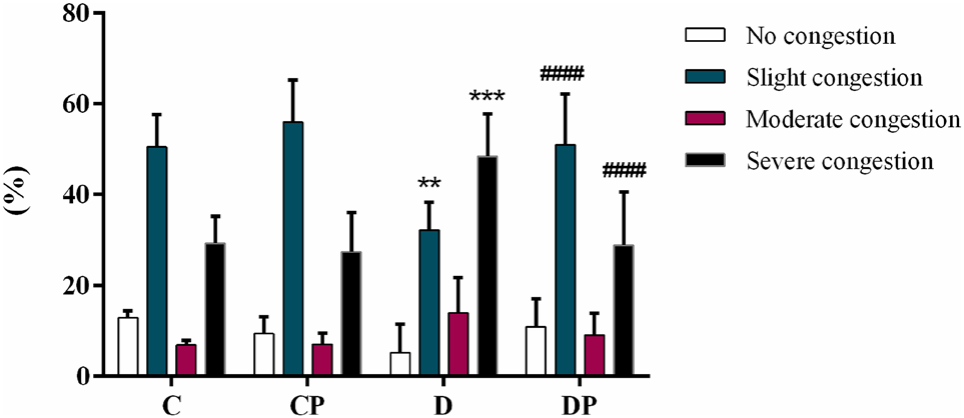
Level of congestion in hepatic vessels (%) from control (C), control + probiotic (CP), diabetic (D) and diabetic + probiotic (DP) groups (n = 6–9/group). Data are from C57BL/6 STZ-induced diabetic mice, + /− *Saccharomyces boulardii* (0.5 × 10^8^ colony-forming units, THT 500101 strain*,* Probiotics and Starters Cultures, Belgium) for 8 weeks. Two-way ANOVA followed by Tukey test for multiple comparisons of means (GraphPad Prism 6). **p ≤ 0.01, ***p ≤ 0.001 versus C in the same degree of injury; and ^####^p ≤ 0.0001 versus D in the same degree of injury.

Regarding the degree of hepatic congestion, C, CP, and DP groups (Fig. 3 a, b, and d, respectively) presented mostly slight congestion. On the other hand, most of the hepatic vessels from non-treated diabetic mice exhibited severe congestion (Fig. 3c). Figure 4 presents the percentage of vessels classified as presenting no congestion, slight, moderate, or severe congestion in the histological analyses of the hepatic tissue, of each group.

#### Hydropic degeneration

**Figure 5:**
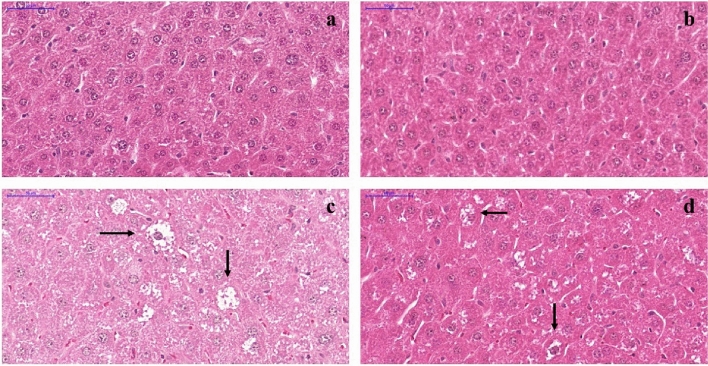
Histological sections of liver stained with Hematoxylin & Eosin from C57BL/6 STZ-induced diabetic mice, + /- *Saccharomyces boulardii* (0.5 × 10^8^ colony-forming units, THT 500,101 strain*,* Probiotics and Starters Cultures, Belgium) for 8 weeks. Control **(a)** and Control treated with *S. boulardii* **(b)** show normal hepatocytes organization. The diabetic group **(c)** showed hepatocytes with signs of hydropic degeneration which consists in enlarged cells and clear cytoplasm due to the presence of small clear vacuoles with indistinct shape and limits (→), and in diabetic group treated with *S. boulardii* **(d)** it is attenuated.

Signs of severe hydropic degeneration were found in hepatic tissue from D group (Fig. 5c). In mice from DP group, however, *S. boulardii* attenuated the injury as shown in Fig. 5d. In mice from C and CP groups the hepatocytes show normal organization (Fig. 5a,b).

### Circulating ALT and AST levels

**Figure 6:**
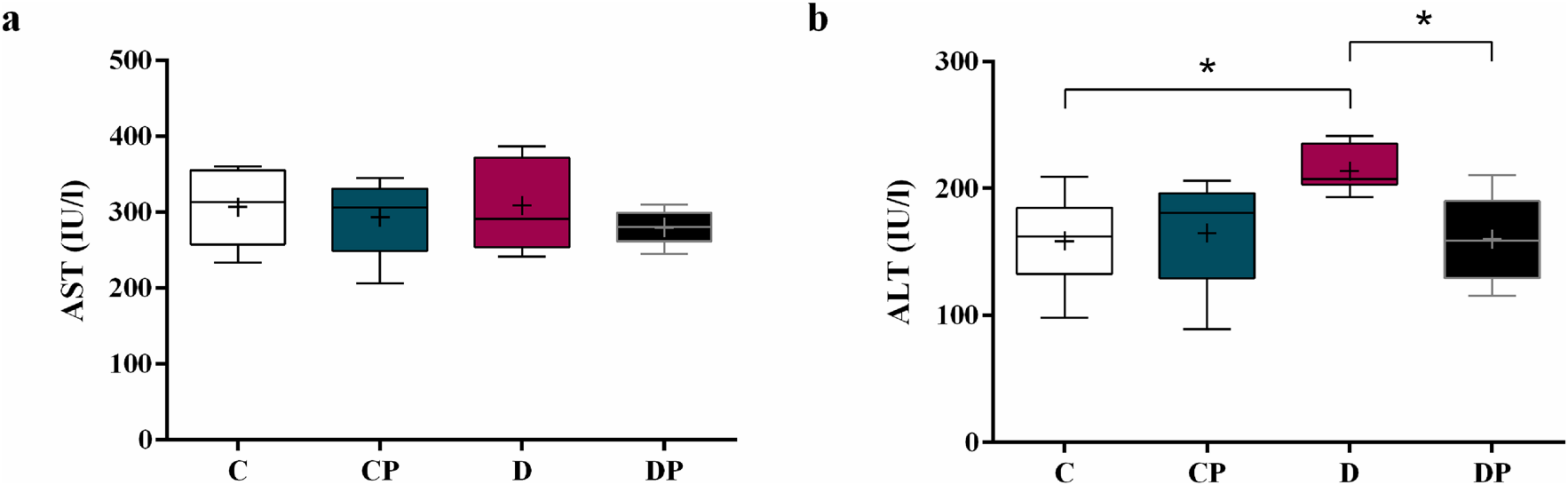
Aminotransferases **(a)** AST and **(b)** ALT in IU/l. Control (C), control + probiotic (CP), diabetic (D) and diabetic + probiotic (DP) groups (n = 6–9/group). Data are from C57BL/6 STZ-induced diabetic mice, + /− *Saccharomyces boulardii* (0.5 × 10^8^ colony-forming units, THT 500,101 strain*,* Probiotics and Starters Cultures, Belgium) for 8 weeks. A horizontal line inside the box indicates the median value of the samples within each group, and the upper and lower edges of the box indicate the quartiles. Two-way ANOVA followed by Tukey test for multiple comparisons of means (GraphPad Prism 6). + indicates mean value. *p ≤ 0.05.

Serum concentration of AST did not significantly change in response to diabetes of probiotic (Fig. 6a). On the other hand, ALT did increase in D group and *S. boulardii* reduced the concentration to the control level (Fig. 6b) (D = 213.7 ± 6.728 IU/l vs. C = 158.4 ± 11.56 IU/l, DP = 159.8 ± 13.83 IU/l).

### Carbonylated proteins and lipid peroxidation

**Figure 7:**
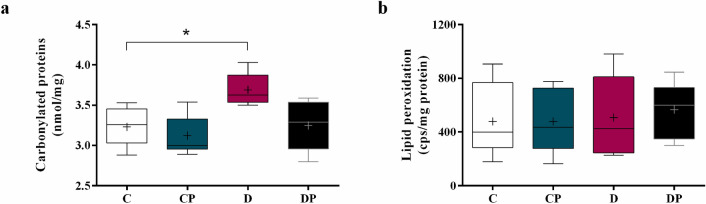
Carbonylated proteins (nmol/mg) and lipid peroxidation (cps/mg protein) in hepatic tissue of control (C), control + probiotic (CP), diabetic (D) and diabetic + probiotic (DP) groups (n = 6–9/group). Data are from C57BL/6 STZ-induced diabetic mice, + /- *Saccharomyces boulardii* (0.5 × 10^8^ colony-forming units, THT 500,101 strain*,* Probiotics and Starters Cultures, Belgium) for 8 weeks. A horizontal line inside the box indicates the median value of the samples within each group, and the upper and lower edges of the box indicate the quartiles. For **(a)** the significance was determined by Kruskal–Wallis followed by Dunn’s test and for **(b)** the significance was determined by Two-way ANOVA followed by Tukey test for multiple comparisons of means (GraphPad Prism 6). + indicates mean value. *p ≤ 0.05.

The measurement of carbonylated proteins showed significantly higher levels of protein damage in D compared to C group (Fig. 7a) (D = 3.62 ± 0.33 nmol/mg vs. C = 3.26 ± 0.42 nmol/mg), and probiotic attenuated this alteration in DP group (DP = 3.29 ± 0.58 nmol/mg). No significant alterations regarding lipid peroxidation were observed among the experimental groups (Fig. 7b).

### Antioxidant enzyme activity

**Figure 8:**
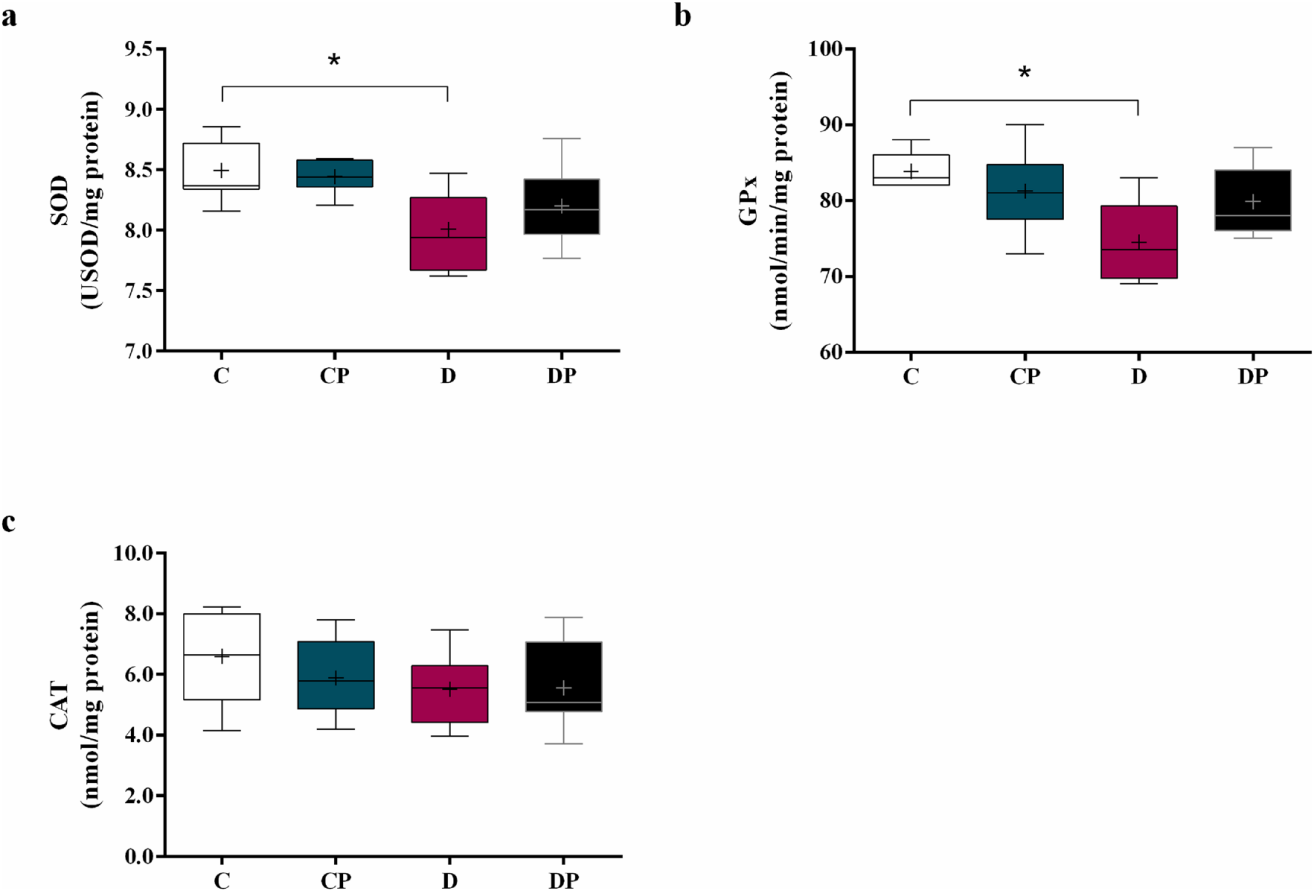
Activity of **(a)** superoxide dismutase (SOD), **(b)** glutathione peroxidase (GPx) and **(c)** catalase (CAT) in hepatic tissue of control (C), control + probiotic (CP), diabetic (D) and diabetic + probiotic (DP) groups (n = 6–9/group). Data are from C57BL/6 STZ-induced diabetic mice, + /- *Saccharomyces boulardii* (0.5 × 10^8^ colony-forming units, THT 500,101 strain*,* Probiotics and Starters Cultures, Belgium) for 8 weeks. A horizontal line inside the box indicates the median value of the samples within each group, and the upper and lower edges of the box indicate the quartiles. For **(a)** significance was determined by Two-way ANOVA followed by Tukey’s test. For **(b, c)** results were evaluated with Kruskal–Wallis, followed by Dunn’s test. + indicates mean value (GraphPad Prism 6). * p ≤ 0.05.

SOD activity decreased in D group compared to C (D = 8.00 ± 0.12 USOD/mg vs. C = 8.49 ± 0.10 USOD/mg). After treatment, SOD activity showed a trend to recover (DP = 8.20 ± 0.14 USOD/mg), as shown in Fig. 8a. GPx presents the same pattern as SOD (C = 83.00 ± 4.00 nmol/min/mg, D = 73.50 ± 9.50 nmol/min/mg, DP = 78.00 ± 8.00 nmol/min/mg) (Fig. 8b). CAT activity was not influenced by diabetes or probiotic, as shown in Fig. 8c.

### Renin angiotensin system components

**Figure 9:**
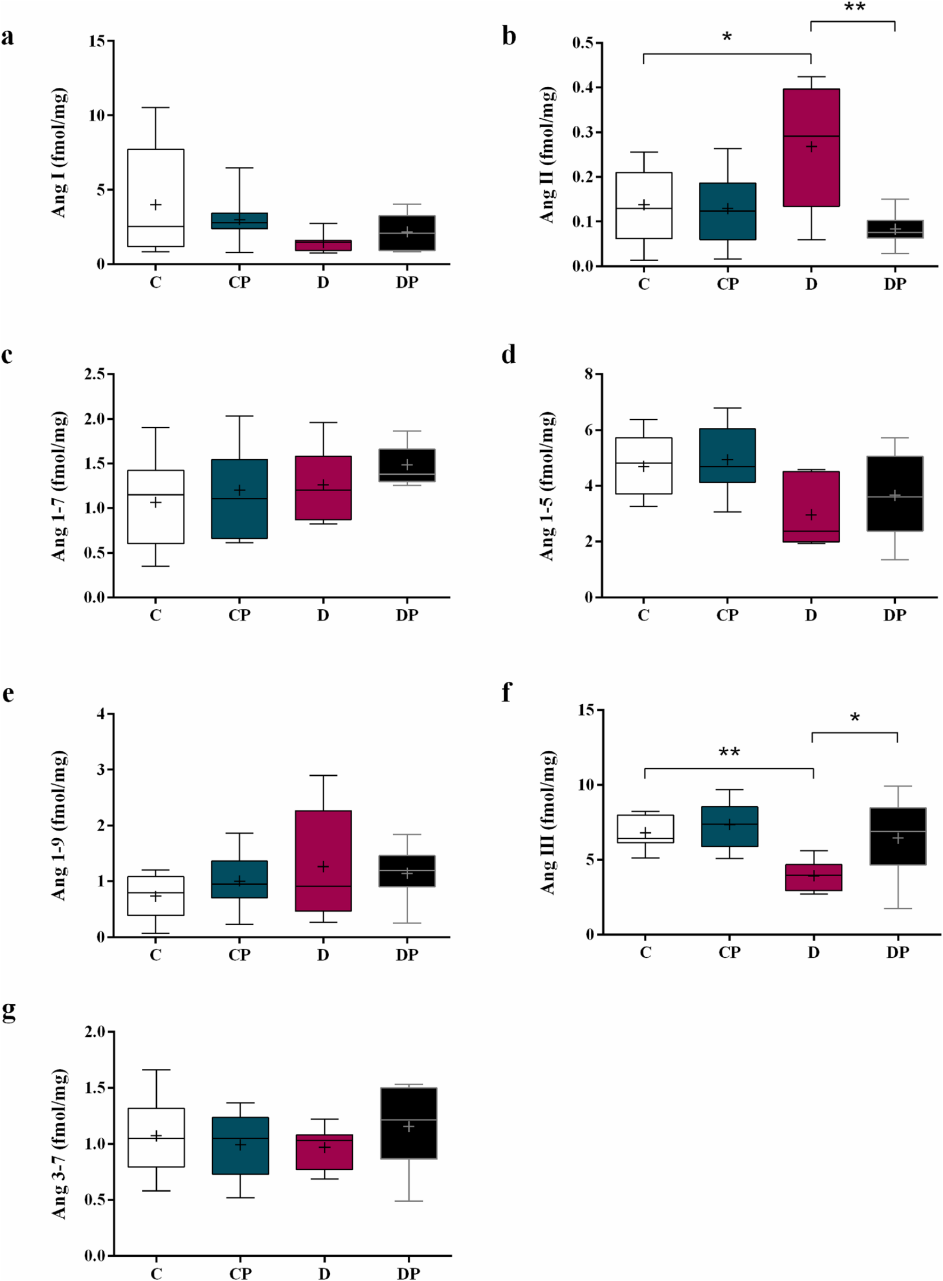
Liver concentration of **(a)** Ang I, **(b)** Ang II, **(c)** Ang (1–7), **(d)** Ang (1–5) **(e)** Ang (1–9), **(f)** Ang III and **(g)** Ang (3–7) in control (C), control + probiotic (CP), diabetic (D) and diabetic + probiotic (DP) groups (n = 6–9/group). Data are from C57BL/6 STZ-induced diabetic mice, + /- *Saccharomyces boulardii* (0.5 × 10^8^ colony-forming units, THT 500101 strain*,* Probiotics and Starters Cultures, Belgium) for 8 weeks. A horizontal line inside the box indicates the median value of the samples within each group, and the upper and lower edges of the box indicate the quartiles. The significance was determined by Two-way ANOVA followed by Tukey’s test for multiple comparisons of means (GraphPad Prism 6). + indicates mean value. *p ≤ 0.05, **p ≤ 0.01.

Liver Ang I, Ang 1–7, Ang 1–5, Ang 1–9, and Ang 3–7 concentrations were not affected by diabetes or treatment (Fig. 9a,c–e,g). On the other hand, liver Ang II increased in D compared to C group. In DP group, Ang II concentration returned to control level (Fig. 9b) (D = 0.27 ± 0.05 fmol/mg vs. C = 0.14 ± 0.02 fmol/mg and DP = 0.08 ± 0.01 fmol/mg). Ang III concentration decreased in D group and the values return to the control level in DP group (Fig. 9f) (D = 3.93 ± 0.35 fmol/mg vs. C = 6.81. ± 0.34 fmol/mg, and DP = 6.45 ± 0.92 fmol/mg).

## Discussion

Some studies have already evaluated the anti-diabetic effects of probiotic strains in animal models^16,34–37^, as well as the close association between the gut microbiota and the development of liver injury^19,21,23,38–40^. Gut microbiota modulation may induce beneficial adaptations, including the reduction of oxidative stress and inflammation, attenuation of fibrosis, and decrease of apoptosis and necrosis^38^. Our research group has already demonstrated the benefits of *S. boulardii* in cardiac function, liver metabolism, and inflammatory profile of diabetic STZ-induced mice. In the present study, we have shown the beneficial effects of *S. boulardii* on liver oxidative stress and local renin angiotensin system, attenuating liver damage on the model of T1DM mice.

Persistent hyperglycemia stimulates the production of reactive oxygen species and decreases the antioxidant defense, increasing liver oxidative stress and also the complications of T1DM^4,41^. In the present study, we demonstrated that diabetes was associated with a decrease of liver antioxidant enzymes SOD and GPx, with no effect on CAT activity, consistent with previous findings^42–45^. We also observed an increased concentration of carbonylated proteins in the diabetic group compared to the control, probably due to an overload on metabolic pathways of reactive carbonyl species detoxification^8^.

On the other hand, diabetic mice treated with *S. boulardii* exhibited a decreased concentration of carbonylated proteins and also a trend to increase the SOD and GPx activities. In this case, the attenuation of hepatic oxidative stress was associated with the improvement of blood glucose control, reducing the formation of free radicals in response to *S. boulardii*. Although the induction of experimental T1DM by STZ causes intense destruction of pancreatic beta cells, drastically reducing the levels of insulin secreted by the pancreas, a previous study has shown that 10—14% of the cells are preserved even when high doses of the substance are used (up to 200 mg/kg)^25^. Thus, in the present study, the reduction of blood glucose and hepatic oxidative stress may be related to the effect of *S. boulardii* on reminiscent beta cell, improving insulin production as previously demonstrated by our group^23^. Moreover, probiotics modulate the redox state by increased antioxidant defenses and modulation of gut microbiota after liver injury^21,23,39,46^, and in fact, it has already been shown that *S. boulardii* presents strong antioxidant activity and reducing power^47,48^.

The activation of hepatic stellate cells, which produce excessively type 1 collagen, is the main factor contributing to liver fibrosis and can be caused by the generation of free radicals and by malondialdehyde, which is a product of lipid peroxidation^49^. Our results show that after eight weeks of treatment there was still no significant increase in the lipid peroxidation, neither stimulation for exacerbated collagen production by hepatic stellate cells in diabetic animals. However, we also demonstrated that the liver of diabetic mice presented vessel congestion compared to control mice, suggesting the presence of fibrotic tissue impairing blood flow. Along with the attenuation of oxidative stress in the treated group, the severe congestion of vessels was reversed after *S. boulardii* administration.

It has been described that the liver presents a local RAS^50^ and that the major effector peptide of the system, Ang II, can be secreted by activated hepatic stellate cells and plays an important role in amplifying oxidative stress in the organ^14,15^. Investigating these local RAS components, our analysis shows that Ang II is increased in diabetic mice and *S. boulardii* reduced the hepatic concentration of this peptide to the control level. On the other hand, the concentration of Ang III was decreased in the diabetic mice compared to the control group, probably as a result of the loss of aminopeptidase A activity. In the present study, the hepatic concentration of Ang II was found lower than the concentration of other RAS peptides, including Ang III. Similar results have already been described in the literature, showing that Ang I and Ang III concentration were higher than Ang II in the plasma of wild type mice^51^. However, it is worth mentioning that the liver of rats with chronic injury are hyper-responsive to Ang II^52^, suggesting that Ang II exerts important effects despite its low concentration in this tissue.

Ang II actions can be mediated by the Ang II receptors (AT1R and AT2R). Ang II/AT1R is the classical RAS, while Ang II/AT2R and Ang (1–7)/MasR are the alternative RAS, and counterbalance the effects of the classical axis. Although we did not evaluate the expression of AT1 and AT2 receptors in the present study, based on our results and those described in the literature, we believe that the activation of the classical RAS (Ang II/AT1R) is at least in part responsible for the liver damage described in the diabetic group, and also that *S. boulardii* exerts some modulation on this pathway. It is well known that both of the Ang II receptors (AT1R and AT2R) are expressed in the liver; however, the AT1R is reported to be far more abundant and responsible for most of the Ang II-mediated effects^53^. Furthermore, Bosnyak showed that all endogenous angiotensin peptide fragments show a higher affinity for AT2R than Ang II, and they suggest the metabolites act mainly through AT2R while Ang II acts through AT1R^54^. Besides, a large number of studies have shown that the activation of the ACE/Ang II/AT1R pathway is associated with various pathological responses including diabetic injury^55^, and as mentioned before, Ang II interaction with AT1R enhances oxidative stress^56^. Gopal et al. showed that Ang II infusion in the liver caused hepatic damage triggering hepatocyte degeneration, hepatic cell apoptosis, sinusoidal dilatation and fluid accumulation^57^. Accordingly, our results show increased oxidative stress in diabetic mice concomitant with increased levels of Ang II and liver damage. On the other hand, *S. boulardii* reduced the Ang II concentration back to the control level, and also attenuated organ damage in diabetic treated groups, suggesting an important role of this peptide on the development of diabetic liver injury.

Based on the histomorphological investigation, we also demonstrated that the number of hepatocytes is decreased in the liver of diabetic mice when compared to the non-diabetic groups, although the liver mass is not reduced. Moreover, we observed that hepatic cells are enlarged in D groups and that the probiotic does not have an influence on any of these parameters. Based on these data, we concluded that as long as hyperglycemia and the consequent increased oxidative stress lead to hepatic cells apoptosis and necrosis, hypertrophy in the diabetic group is occurring as a compensatory mechanism for organ mass loss. Besides that, as the angiotensin generated peptide fragments are also associated with cell growth, cell proliferation, and apoptosis^14,58^, the increased level of Ang II in the liver as well as the prolonged stimulation of the system, are contributing to the abnormal cell growth^59,60^.

Consistent with our finds, studies showed that STZ-induced diabetic rodents presented congestion in portal vessels and sinusoids and hydropic degeneration of hepatocytes in the analysis made between two and eight weeks after the induction^61, 62^. The hydropic degeneration, also evidenced in the present study, is caused by the retention of electrolytes and water by cells as a result of an imbalance in their transport across membranes. This imbalance, in turn, can result from injuries to the plasma membrane caused by oxidative stress. The retention of sodium, the decrease in the amount of potassium, and the consequent increase of intracellular osmotic pressure lead to water influx, and cells acquire a vacuolar aspect^63^. Together with the decrease in oxidative stress markers, hydropic degeneration was attenuated after the administration of *S. boulardii.* Although hydropic degeneration could contribute to cell enlargement in the context of diabetes, it does not seem to be the main factor, since the administration of *S. boulardii* attenuates this maladaptation with no significant effect on liver mass. We hypothesize that *S. boulardii* attenuates hydropic degeneration induced by diabetes, but not the mechanisms responsible for cell hypertrophy. Our results are consistent with others showing that to survive oxidative stress, hepatic cells may inhibit proliferation, and the recovery of liver mass preferentially occurs by hypertrophic mechanisms, allowing immediate responses to maintain homeostasis^64–66^. It is important to mention that although we did not observe hepatic steatosis in this study, Albuquerque et al. demonstrated that the content of triglycerides is increased in STZ-induced diabetic mice and that the treatment with *S. boulardii* restored it.

At last, we can affirm that the non-diabetic mice receiving probiotic *S. boulardii* did not present a considerable difference when compared to control in any of the evaluated parameters, which proves that the probiotic administration does not have any effect on oxidative stress, RAS or cause damage to the liver of healthy animals. Our results show that neither AST nor ALT levels were negatively affected by *S. boulardii* administration. On the other hand, increased ALT activity was observed in D group, and *S. boulardii* reduced it, showing that the hepatocellular injury induced by diabetes was attenuated by the probiotic. Aminotransferases AST and ALT are both found in high concentrations in hepatic cells, and they leak into the circulation when the hepatocytes are damaged^67,68^. AST can also be found in the heart muscle, skeletal muscle, kidney, neuronal cell bodies, pancreas and blood cells, while ALT is primarily in the liver^69,70^. Therefore, an increase in ALT is a more specific indicator of liver injury. Moreover, as the intracellular ALT is found only in the cell cytoplasm, while most of AST content is found in the cell mitochondria, increased AST is related to severe hepatic injury since it shows not only cytoplasmic enzymes leaked into the circulation but the mitochondrial enzymes as well^70,71^.

Limitations of the present study include the lack of expression analysis of the angiotensin receptors (AT1R and AT2R) in the liver. The precise pathways activated by Ang II and Ang III in the context of diabetes and *S. boulardii* treatment remain to be elucidated, but the results from the present study do imply that the imbalance of RAS peptides could well have a role in the development of diabetic-liver injury. Besides, our results demonstrated that *S. boulardii* administration normalizes the concentration of RAS peptides while liver damage was also attenuated, showing that the changes in RAS peptides levels are at least in part responsible for this beneficial effect. Further studies will be needed to elucidate which pathway is predominantly activated by Ang II and Ang III, to understand the exact function of those peptides in the liver of diabetic animals.

Thus, in summary, this study provided histomorphological and biochemical analyses on the liver after *S. boulardii* administration on T1DM mice. Our results demonstrated that *S. boulardii* administration in STZ-induced diabetic mice reduces oxidative stress, normalizes the level of RAS components, and attenuates diabetes-induced liver injury, supporting the hypothesis that this yeast may have a role in the treatment of diabetes as a potential adjunctive therapy to reduce hepatic and metabolic complications of the disease.

## Data Availability

The datasets generated during and/or analyzed during the current study are available from the corresponding author on reasonable request.
